# Promoting the use of social networks in pneumonia

**DOI:** 10.1186/s41479-020-00066-3

**Published:** 2020-05-25

**Authors:** Catia Cillóniz, Leith Greenslade, Cristina Dominedò, Carolina Garcia-Vidal

**Affiliations:** 1 August Pi i Sunyer Biomedical Research Institute – IDIBAPS, University of Barcelona, C/ Villarroel 170, 08036 Barcelona, Spain; 2 Biomedical Research Networking Centers in Respiratory Diseases (CIBERES), the Association of Support and Information for Family members and Patients with Pneumonia (NEUMOAI), Barcelona, Spain; 3 Every Breath Counts Coalition, New York City, USA; 4 Department of Anesthesiology and Intensive Care Medicine, Fondazione Policlinico Universitario A. Gemelli, Università Cattolica del Sacro Cuore, Rome, Italy; 5 Department of Infectious Diseases, Hospital Clinic of Barcelona, Barcelona, Spain

**Keywords:** Pneumonia, Social networks, Education, Pneumolight, Awareness

## Abstract

**Background:**

Pneumonia is a serious health concern, but it does not attract the attention it warrants. Perhaps this is due to a lack of understanding of the real extent of this infectious disease in the general population.

**Methods:**

A literature review was performed to assess the role of social networks as a means to raise awareness over pneumonia worldwide and increase its visibility.

**Results:**

In 2017, approximately 800,000 children under 5 years and approximately one million older people died of pneumonia. The importance of this pathology remains underestimated, despite the publication of many articles, comments, and editorials dedicated to rectifying the imbalance and to reduce its impact and associated mortality. Current misperceptions about pneumonia are alarming. Education and awareness are essential in the fight against this major public health threat; in this endeavor, social networks can be used to distribute science-based information about the disease and thus raise awareness among the general public about the dangers it poses. Approximately 3.8 billion people were using social media at the beginning of 2020, representing more than half of the world’s population.

**Conclusion:**

Social networks offer a valuable tool for disseminating scientific information about pneumonia, increasing its visibility, and in general raising awareness about this preventable disease.

## Social networks and science: why do they have so much power?

Social networks are powerful engines that can capture information and deliver immediate feedback that is free of charge. Currently, a large numbers of the world’s population uses general social networks such as Twitter, Facebook, YouTube, Instagram and LinkedIn, with many also using specialist networks like ResearchGate [1]. Having incorporated these networks into daily lives, a sizable proportion of people will use them to search for healthcare information, to guide self-diagnosis or to help diagnose others. People with chronic diseases are especially likely to use social networks to learn more about their condition, improve active participation in treatment-related decisions, and also to seek peer support. However, the benefits of social networks are not limited to these aspects information and expression. They have become very useful to the scientific world, providing a route to promote scientific culture, to teach, to improve skills, to improve visibility, and to communicate science to wider society.

**Fig. 1:**
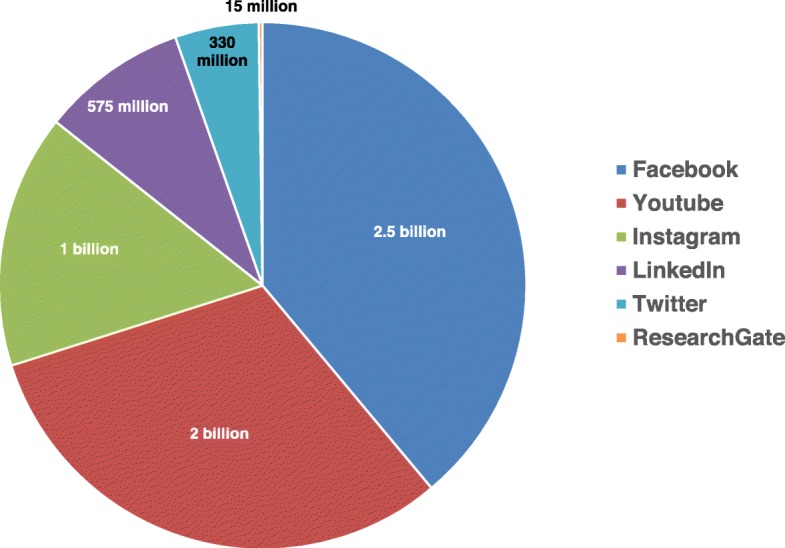
Numbers of users globally according to social network

According to the Digital 2020 report by Simon Kemp [2], at the beginning of 2020 approximately 4.5 billion people worldwide were using Internet and 3.8 billion were using social media, representing more than half of the world’s population. A 2019 report from the UK about the use of social media, revealed that 93% of people aged between 16 and 24 years have a social media profile compared with 20% of adults aged 70 years and older [3]. The numbers of users varies according to social network, with 2.5 billion for Facebook, 2 billion for YouTube, 1 billion for Instagram, 575 million for LinkedIn, 330 million for Twitter, and 15 million for ResearchGate (Fig. 1). On average, an internet user spends 2 h and 4 min using social media across all devices in a day.

## The burden of pneumonia

**Fig. 2:**
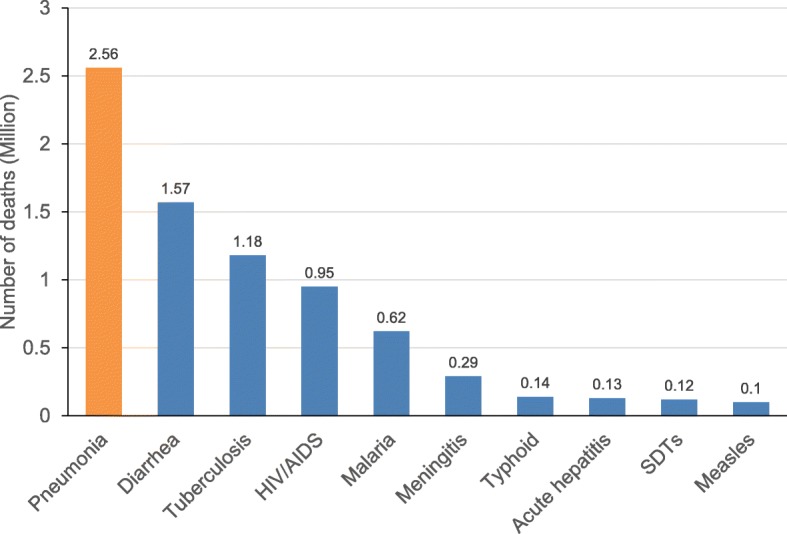
Pneumonia is the leading infectious cause of death in the world (source Global Burden of Disease, 2017)

**Fig. 3:**
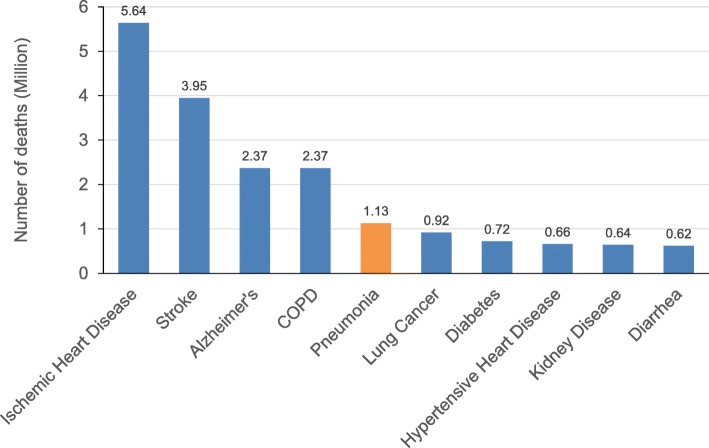
Pneumonia is the fifth leading cause of death among adults over 70 (source Global Burden of Disease, 2017)

The incidence and mortality of pneumonia are high [4, 5]. Epidemiological data indicates that pneumonia remains a neglected disease and the leading infectious cause of death worldwide (Fig. 2), especially among vulnerable groups, including children (aged < 5 years) and older people (aged > 70 years) [4, 6, 7]. In 2016, the Global Burden of Diseases, Injuries, and Risk Factors Study [8] showed that lower respiratory tract infections affected 336.4 million people globally, with an estimated 65.9 million hospitalizations among all age groups and 2.3 million deaths. Despite a reported decrease of 36% in total deaths from lower respiratory tract infections between 2007 and 2017 among children (aged < 5 years), there was a reported increase of 34% among older adults (aged > 70 years) the following year [9]. A closer look reveals that approximately 800,000 children younger than 5 years and 1 million people older than 70 years died because of pneumonia in 2017 (Fig. 3).

However, pneumonia is not limited to these age groups and affects all ages, with pneumonia-related deaths occurring in 44,000 children aged 5 to 14 years, 170,000 people aged 15 to 49 years, and 406,000 adults aged 50 to 69 years in 2017 [9].

## The role of social media as a platform for science communication

Pneumonia is an underestimated and neglected disease [4]. Given the benefits of social networks, we contend that cyberspace offers immense potential to improve the visibility and raise awareness of this major clinical and public health problem, among both the general population and healthcare professionals. Social networks offer a major opportunity to provide education, improve visibility, and transform the perception of pneumonia worldwide.

Social media platforms have been used with success in a variety of areas of scientific communication. Among these, the following stand out: disseminating scientific information [10, 11], expanding professional connections, exploring new research, launching research projects and grant calls, promoting conferences [12, 13], facilitating professional and research networking [14], changing scientific experience, providing healthcare education [15] and training, improving the visibility of professional health associations, and increasing the audience of scientific journals [16, 17]. Of course, social networks in the scientific world should complement, and not replace, traditional modes of scientific communication, such as peer-reviewed journals.

A survey published in 2014 by *Nature* explored the scientific and social media participation of more than 3000 professionals [1]. Approximately 50% of them declared that they used social media to follow discussions and post work content; 40% used social media to discover peers and recommended papers, to comment on research, and to share links to authored content; finally, 20% used social media to discuss research, discover jobs, for case contact, or for non-professional reasons. Fewer than 10% used social media out of either curiosity or to track metrics. The most frequently visited social media sites in that survey were ResearchGate, LinkedIn, Facebook, Twitter, Academia.edu, and Mendeley.

A study published at the time of the 2009 H1N1 Influenza A pandemic evaluated the information disseminated on Twitter during the pandemic (infoveillance). Specifically, they monitored the use of the terms “H1N1” versus “swine flu” over time and, performed a content analysis of the *tweets* [18]. Over a period of approximately 8 months (from May 1 to December 31), the authors reported over 2 million Twitter posts with the terms “swine flu,” “swineflu,” and/or “H1N1” using the Infovigil system. Based on their findings, they were able to validate Twitter as a tool for monitoring trends in content, feeling, and public attention in real time. They also observed that the proportion of tweets using the term “H1N1” increased compared to the use of the term “swine flu,” which demonstrated that the general public and the media adopted the terminology recommended by the World Health Organization. Approximately 90% of the information on Twitter provided reference to the information shared, allowing for confirmation of the information, and only 4.5% of the manually coded tweets were classified as possible misinformation or speculation, with most information disseminated to the public from reliable sources. This study highlights the potential and feasibility of using social networks to conduct “infodemiology” studies on public health issues. Moreover, information on Twitter can be used for the analysis of feelings, content, and knowledge translation, almost in real time. In turn, this would improve the awareness of health authorities to the concerns of the general public.

The Spanish Society for Microbiology (SEM) conducted an interesting experiment in 2016 to analyze the use of Twitter as a tool for communication about a basic 28-lesson microbiology course over 20 h (10 weeks with classes 3 days a week) [19]. A total of 30 professionals were involved, all members of the SEM, and the *hashtag* #microMOOCSEM was used for promotion. The course was followed in Spain, as well as in Mexico, Venezuela, Argentina, Colombia, Perú, Ecuador, and Chile. College and high school students, high school and higher education teachers, healthcare and science professionals, journalists, and scientific communicators were the most representative of the followers. To assess the acquisition of knowledge, the course included three or four quiz questions at the end of each class, with a total of 78 questions. More than 90% correctly answered 16 questions and more than 50% correctly answered 62 questions. The authors concluded that Twitter provided an excellent tool for teaching and active learning.

## Pneumonia in social media: where are we now?

In 2009, World Pneumonia Day was established on November 12 by the Global Coalition against Child Pneumonia to raise awareness of pneumonia, “the forgotten killer of children” [20]. The aim of this annual day is to promote interventions designed to protect, prevent, and treat pneumonia, as well as to highlight proven approaches and solutions that need additional resources and action (e.g., donor investment). In 2009, pneumonia was killing an estimated 1.2 million children every year. Unfortunately, more than a decade later, pneumonia continues to be a neglected disease, with approximately 800,000 pneumonia deaths in children under 5 years (and 1 million deaths in older people) in 2017 [5]. Several calls to action have been published about pneumonia in the last 10 years to improve the diagnosis, treatment, and prevention of pneumonia worldwide, but there has been limited progress in reducing both pneumonia incidence and mortality [4, 6, 7, 21–25].

In 2017, the Every Breath Counts Coalition (https://stoppneumonia.org/) was launched to provide a strong platform for governmental and non-governmental actors to collaborate at national, regional, and global levels in the fight against child pneumonia deaths, prioritizing the world’s most vulnerable children. The Coalition introduced the hashtags #StopPneumonia and #EveryBreathCounts and was active on Twitter and LinkedIn sharing content hosted on www.stoppneumonia.org. In 2018, “The Missing Piece: why continued neglect of pneumonia threatens the achievement of health goals” report was published on World Pneumonia Day and warned that to reduce the incidence of pneumonia and its associated mortality, important changes were required. These included the need for a “life-cycle” approach to pneumonia control targeting interventions to the most vulnerable populations, including children and the elderly, and special efforts to reduce risk factors for pneumonia death, improve prevention, diagnosis and treatment services.

In 2019, Adnan et al. [26] published a study of the content of tweets with a pneumonia hashtag for World Pneumonia Day from 2011 to 2016, seeking to investigate whether the *tweets* mentioned topics that coincided with the three objectives described in the World Pneumonia Day report of 2011 [27]. In total, 28,181 original tweets including #pneumonia were analyzed during the period of interest, and the top ten users who tweeted #pneumonia were @ExpatInc, @Stop_Pneumonia, @Breathingmatter, @Exelume, @PPT_Search, @USAIDGH, @gavi, @atscommunity, @FightingMalaria, and @ironorehopper. The authors reported that tweets that raised awareness, promoted interventions, or mentioned vaccine costs were more likely to be shared than tweets sharing personal experiences of pneumonia, concluding that the promotion of pneumonia-related health through Twitter around World Pneumonia Day attracted the attention of a large proportion of the general public and global health advocates, which could help future interventions especially by having consider people’s main concerns. Social networks like Twitter provide a useful platform through which public health professionals can raise awareness, distribute evidence-based information, and mobilize supporters to take relevant measures on pneumonia.

Another interesting conference paper was presented at the second international conference on data science and social research [28]. The authors constructed a global health network using the official Twitter accounts of organizations that fight against tuberculosis, malaria, and pneumonia and created a unique and common database for social network analysis. The aim was to assess the structure of the network and the role and performance of each organization. The authors reported that the tuberculosis network had the largest impact, having 1838 nodes and a partial overlap with the HIV/AIDS network. The malaria network was the second in terms of impact, with 196 nodes, while the pneumonia network had the least impact, with 65 nodes. Interestingly, the network density was inversely proportional to the size of the network (the pneumonia, malaria, and tuberculosis networks have densities of 7.3, 1.7, and 0.2%, respectively). The authors also analyzed the geographical cover of these three networks, finding that the tuberculosis network had the higher geographical coverage, followed by the malaria network and then the pneumonia network. This study emphasized the importance and scope of social networks in health issues.

In January of this year, the first Global Forum on Childhood Pneumonia was hosted in Barcelona, an event in which approximately 350 people from 55 countries participated. This event aimed to place pneumonia at the forefront of national and global health agendas, raising awareness of the magnitude of the challenge posed by pneumonia and making it a priority global health concern [29]. The hashtags related to this event were #StopPneumonia, #FightingForBreath, and #EveryBreathCounts. During the 3 days of the global forum, #StopPneumonia was used 5630 times, of which almost 1000 were from original tweets. Moreover, they covered a wide geographical distribution, with usage in Spain, Nigeria, United States, United Kingdom, Uganda, India, Switzerland, Kenya, Canada, Austria, Pakistan, Indonesia, France, Nepal, and Somalia being the most common. Tweets from the Every Breath Counts Twitter handle, @StopPneumonia, generated 271,200 earned impressions during the event. On January 29, the first day of the global forum, #StopPneumonia was trending in Spain. This showed the importance of social networks in reaching a wide global audience increasing the visibility of an event such as the Global Forum on Childhood Pneumonia.

## Social media as a tool for promoting awareness and education about pneumonia

Pneumonia is a serious health concern that does not attract the attention it warrants. Perhaps this is due to a lack of understanding about the extent of the disease, its complications, and mortality, the short- and long-term consequences, as well as the fact that its true incidence may be underestimated. The issues surrounding pneumonia remain neglected despite many articles, comments, and editorials dedicated to rectifying the imbalance and meaningfully change the associated impact and mortality. Examples include “The forgotten killer of children” [20], “Pneumonia: a global cause without champions” [29], “The disgraceful neglect of childhood pneumonia” [30], and “The Missing Piece: why continued neglect of pneumonia threatens the achievement of health goals” [4], each of which shared the common and forceful message that the status quo must change. By bringing greater global visibility to pneumonia we could prevent the needless deaths of 11 million children in the next decade.

**Fig. 4:**
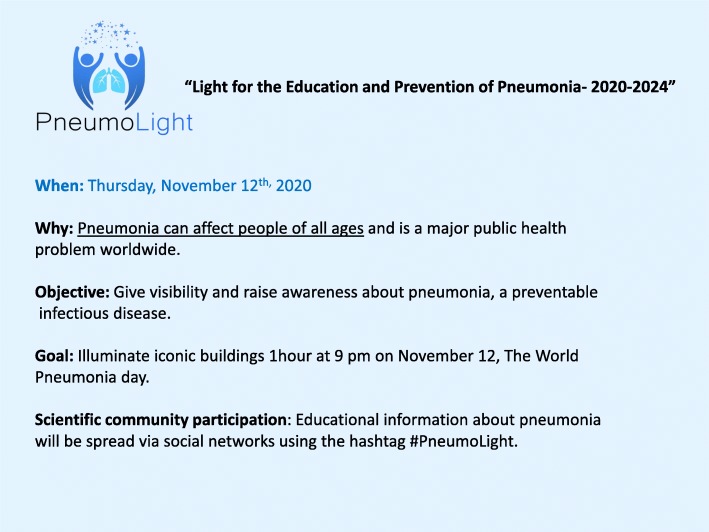
Global Pneumolight Initiative

Education and awareness are important in the fight against pneumonia. The misperceptions about pneumonia and the limited public knowledge, especially about its impact on general health, are alarming. To correct this situation, a new initiative called PneumoLight was started (#PneumoLight) under the coordination of the Association of Support and Information for Family members and Patients with Pneumonia (NEUMOAI) (@neumoai) (Fig. 4). This initiative has also been incorporated into the Every Breath Counts Coalition to contribute to the global fight against pneumonia. PneumoLight, “Light for the Education and Prevention of Pneumonia,” is a non-profit collaborative project that was committed to increase the visibility of pneumonia worldwide and to raise public awareness of its risks. The ability to identify early pneumonia symptoms and the readiness to seek emergency medical care are crucial to access treatment early, especially among at-risk groups (e.g., young children, the elderly, and people with co-morbidities). PneumoLight has set out to bathe prominent buildings in blue light on World Pneumonia Day each year. By increasing the visibility of pneumonia, literally, it will allow educate the public and spread scientifically proven data on this disease. PneumoLight will also engage the global scientific community, responding to questions from the general public and health practitioners about all aspects of pneumonia. Key areas include pneumonia management, main risk groups, short- and long-term complications, mortality, rehabilitation, and preventive measures. Educational information about pneumonia will be spread via social networks using the hashtag #PneumoLight.

The PneumoLight project is operating with the collaboration of several scientific societies and organizations. These include the Forum of International Respiratory Societies (FIRS), the European Lung Foundation (ELF), the European Respiratory Society (ERS), the Institute of Global Health of Barcelona (ISGLOBAL), the Spanish Society of Infectious Diseases and Clinical Microbiology (SEIMC), the Spanish Society of Primary Care Physicians (SEMERGEN), the Spanish Society of Intensive Medicine, Critics and Coronary Units (SEMICYUC), the Valencian Society of Pneumology (SVN), the Catalan Society of Infectious Malalties i Clinical Microbiology (SCMINMC), the Hospital Clinic de Barcelona, the University of Barcelona (UB), the Castellano-Manchega Society of Respiratory Pathology (SOCAMPAR), ​​the Peruvian Society of Internal Medicine (SOPEMI), and the FFPatients Association. This wide involvement is important lest we forget that pneumonia is a disease or health problem that affects many medical disciplines, such as pulmonary or respiratory, primary care, infectious diseases, clinical microbiology, internal medicine, or critical care. Indeed, although pneumonia primarily affects the lungs, it often becomes a systemic infection that can have many short- and long-term complications. Used correctly, social networks can facilitate international cooperation and the dissemination of information in the fight against pneumonia, while also increasing education and awareness more broadly.

## Conclusions

Social networks can play a crucial role in pneumonia knowledge and management. They can be used as tools for distributing science-based information and for increasing awareness of a public health threat that is currently underestimated. We encourage scientific societies, patient associations, foundations, and organizations involved in the fight against pneumonia to use the hashtags #StopPneumonia, #EveryBreathCounts and #Fightingfor Breath and to support new initiatives like #PneumoLight on social media. This social media engagement offers a platform for disseminating and monitoring scientific information on pneumonia. As social media are now a global phenomenon, their use can bring information to the general public in almost every country. Global health advocates thus have a readily available tool that they can use to raise awareness and to promote interventions related to pneumonia. However, it is vital to ensure that the information about pneumonia on social networks has a scientific basis.

## Data Availability

Data sharing is not applicable to this article as no datasets were generated or analysed during the current study.

## Abbreviations

SEM: Spanish Society for Microbiology
NEUMOAI: Association of Support and Information for Family members and Patients with Pneumonia
FIRS: Forum of International Respiratory Societies
ELF: European Lung Foundation
ERS: European Respiratory Society
ISGLOBAL: Institute of Global Health of Barcelona
SEIMC: Spanish Society of Infectious Diseases and Clinical Microbiology
SEMERGEN: Spanish Society of Primary Care Physicians
SEMICYUC: Spanish Society of Intensive Medicine, Critics and Coronary Units
SVN: Valencian Society of Pneumology
SCMINMC: Catalan Society of Infectious Malalties i Clinical Microbiology
UB: University of Barcelona
SOCAMPAR: Castellano-Manchega Society of Respiratory Pathology
SOPEMI: Peruvian Society of Internal Medicine
