# Correction: Promoting the use of social networks in pneumonia

**DOI:** 10.1186/s41479-023-00111-x

**Published:** 2023-04-21

**Authors:** Catia Cillóniz, Leith Greenslade, Cristina Dominedò, Carolina Garcia-Vidal

**Affiliations:** 1 August Pi i Sunyer Biomedical Research Institute – IDIBAPS, University of Barcelona, C/ Villarroel 170, 08036 Barcelona, Spain; 2 Biomedical Research Networking Centers in Respiratory Diseases (CIBERES), the Association of Support and Information for Family Members and Patients With Pneumonia (NEUMOAI), Barcelona, Spain; 3 Every Breath Counts Coalition, New York City, USA; 4 Department of Anesthesiology and Intensive Care Medicine, Fondazione Policlinico Universitario A. Gemelli, Università Cattolica del Sacro Cuore, Rome, Italy; 5 Department of Infectious Diseases, Hospital Clinic of Barcelona, Barcelona, Spain

**Correction: Pneumonia 12, 3 (2020)**

**https://doi.org/10.1186/s41479-020-00066-3**

Following publication of the original article [1], the authors reported an error in the Funding section.

The Funding section currently reads:

**Funding**

No funding.

The Funding section should read:

**Funding**

Funded by Instituto de Salud Carlos III (ISCIII) through the project FIS PI18/01061 and co-funded by the European Union.

The funding section has been updated above and the original article [1] has been corrected.
